# Manual for Hospital Nurses

**Published:** 1885-10

**Authors:** 


Manual for Hospital Nurses and Others Engaged in Caring for the Sick. *By* Edward J. Domville, m. d. *Fifth edition, pp. 96. Philadelphia: P. Blakiston, Son & Co. Chicago: W. T. Keener.*

That a fifth edition of this little book is demanded is sufficient evidence that it has been found useful. It contains a brief outline of the nurse's duties and suggestions as to her deportment.

Section I, upon the care of ordinary cases, is intended to prepare the nurse for all the usual duties of the sick-room—such as management of temperature, ventilation, the care of the patient's person, the administration of food and medicine.

Section II, upon extraordinary cases, gives directions for procedure in accidents and emergencies, in operations, and for the care and treatment of special cases.

An appendix is devoted to the preparation of remedies and diet for the sick, a table of weights and measures, a list of medical abbreviations, and a glossary of common medical terms. F. S. J.

